# Influence of Spark Plasma Sintering Parameters on the Microstructure, Mechanical and Tribological Characteristics of Air-Milled Aluminum

**DOI:** 10.3390/ma18245652

**Published:** 2025-12-16

**Authors:** Hanen Ammari, Sophie Le Gallet, Pierre-Henri Cornuault, Frédéric Herbst, Nicolas Geoffroy, Mahmoud Chemingui, Virgil Optasanu

**Affiliations:** 1 Laboratory of Inorganic Chemistry, LR 17-ES-07, University of Sfax, B.P. 1171, Sfax 3018, Tunisia; hanen.ammari@u-bourgogne.fr; 2 Laboratoire Interdisciplinaire Carnot de Bourgogne—ICB UMR 6303 CNRS, Université Bourgogne Europe, B.P. 47870, F-21078 Dijon, France; sophie.le-gallet@u-bourgogne.fr (S.L.G.);; 3 Université Marie et Louis Pasteur, UFR ST, CNRS, Institut FEMTO-ST, F-25000 Besançon, France; pierre_henri.cornuault@univ-fcomte.fr

**Keywords:** aluminum, high-energy ball milling, spark plasma sintering, microstructure, tribology, mechanical properties

## Abstract

This work investigates the influence of spark plasma sintering (SPS) parameters on the microstructure and mechanical properties of consolidated aluminum powders processed by high-energy ball milling under an air atmosphere. Sintering was performed under vacuum at various temperatures ranging from 550 °C to 625 °C and under pressures between 50 and 100 MPa. The particle size, crystallite size, and microstructure of the powders and the consolidated pellets were analyzed using laser granulometry, X-ray diffraction (XRD), scanning electron microscopy (SEM), and Archimedes’ density measurements. Mechanical properties were evaluated via Vickers microhardness, nanoindentation, and tribological testing. For comparison, unmilled aluminum powders were also consolidated and characterized. After 46 h of milling, the aluminum crystallite size was reduced from 74 nm to 68 nm. The sample’s density increased with higher sintering temperature and pressure. The aluminum sintered at 600 °C and 100 MPa after 46 h of milling exhibited the highest microhardness (187.5 HV). Nanoindentation tests were conducted to characterize different microstructural regions formed after SPS, revealing two distinct zones: one hard and one soft. The tribology results revealed that the SPS-consolidated samples of milled powders exhibited a reduction of 50% in specific wear rate and a reduction of 20% in the coefficient of friction compared to the SPS-sintered samples of unmilled powders.

## 1. Introduction

Aluminum and its alloys are at the center of extensive research due to their excellent combination of properties, including a high strength-to-weight ratio, broad ductility and stiffness range, low density, enhanced fatigue and wear resistance, corrosion resistance, thermal stability, and excellent electrical conductivity [1,2,3]. Because of these attributes, aluminum is widely used across various sectors, including defense, electrical engineering, aerospace, automotive, and construction industries [4,5,6].

However, the relatively low strength of aluminum alloys remains a significant limitation in expanding their structural applications [7,8]. According to the Hall–Petch approach, material strength increases with decreasing grain size [9], as a higher density of grain boundaries impedes dislocation motion. This microstructural refinement improves mechanical properties such as strength, hardness, and wear resistance. Enhancing the mechanical performance of aluminum continues to be a key research focus.

Mechanical alloying (MA), especially through high-energy ball milling, is a promising technique to reduce grain size to the nanometric scale [10]. Initially developed by Benjamin [11] for oxide dispersion-strengthened nickel-based superalloys, MA involves two primary mechanisms: powder fracturing and cold welding [12]. Fracturing breaks down grains into finer domains, increasing strength through grain refinement and dislocation accumulation. Cold welding facilitates the agglomeration of particles into clusters [13]. By adjusting milling parameters such as speed and duration, various powder morphologies can be achieved with significantly reduced grain sizes. While this method enhances strength through a high density of crystallographic defects, it can also decrease ductility and promote brittleness. Additionally, the heat generated during milling contributes to nano-grain restructuring, increased defect mobility, and partial oxidation of aluminum, especially under an air atmosphere.

The milled powders can be consolidated using various powder metallurgy processes such as hot pressing (HP), hot extrusion, and metal injection molding. More advanced methods like hot isostatic pressing (HIP) and spark plasma sintering (SPS) were recently developed. Although molding, HIP, and extrusion offer good formability, they are often cost-prohibitive. Furthermore, prolonged exposure to high temperatures during these processes can induce undesirable grain growth and dislocation annihilation, negatively impacting mechanical properties such as strength and wear resistance [14]. SPS has emerged as a particularly efficient technique for consolidating milled powders while retaining their refined microstructure. This is due to its rapid heating rates and short dwell times, which limit grain coarsening and the formation of undesired phases [15,16,17,18]. SPS has been successfully used to consolidate a wide variety of metals, ceramics, and alloys [19].

Several studies have explored the effect of SPS parameters on aluminum. Xie et al. [20] examined the effect of pressure (9.4 MPa, 23.5 MPa, 47 MPa) and temperature (300 °C, 350 °C, 400 °C, 500 °C, and 600 °C) on the microstructure of commercial pure aluminum unmilled powder consolidated by SPS. They found that higher sintering temperature and pressure (600 °C and 47 MPa) improved particle bonding, reduced electrical resistivity, and enhanced tensile strength. Liu et al. [21] studied nanocrystalline aluminum powders consolidated by SPS at 400–600 °C under 300 MPa pressure. They observed a decrease in intergranular porosity and reported peak mechanical performance (3 GPa microhardness, 665 MPa compressive strength, 282 MPa tensile strength) after SPS consolidation at 500 °C.

Mohammed et al. [22] investigated the tribological properties of pure aluminum milled for 24 h and SPS-consolidated at 550 °C and 50 MPa–10 min, reporting a coefficient of friction of 0.85 and a wear rate of 6 × 10^−4^ mm^3^/(N·m) using stainless steel balls as counter face and 3 N load. Rengifo et al. [23] reported a coefficient of friction of 0.87 and a wear rate of 5.7 × 10^−3^ mm^3^/(N·m) for raw unmilled powder Al consolidated by SPS at 500 °C–50 MPa–10 min. The tribological tests were made under a 1 N normal load against an Al_2_O_3_ ball with a diameter of 3 mm as a counter face. Tabandeh-Khorshid et al. [24] investigated the effect of increased sliding speed and normal loading on the tribological properties of pure Al milled for 6 h and consolidated by cold compaction at 200 MPa, followed by hot compaction at (500 MPa, 525 °C), using 5 mm diameter pins made of consolidated Al powder on stainless steel disks. They found a coefficient of friction of 0.35.

All the cited studies have used mechanical alloying processes under an Ar atmosphere to prevent partial oxidation of Al. This makes the industrialization of such processes substantially more complicated than the use of milling under an air atmosphere. The simplification of the high-energy milling procedure would be interesting to evaluate. In this study, pure aluminum powder was mechanically milled for 46 h using high-energy ball milling under an air atmosphere. The powders were then consolidated by SPS at various temperatures and pressures. This research focuses on three primary objectives:

- Investigating the effect of mechanical milling on the powder and sintered sample microstructure.
- Evaluating the influence of sintering temperature and pressure on the density and microstructure of both milled and unmilled aluminum.
- Assessing how sintering parameters and microstructure affect tribological and mechanical properties such as hardness and elastic modulus.

Finally, the experimental results are critically compared with findings from the literature to provide comprehensive insight into the processing–structure–property relationships in SPS-consolidated aluminum.

## 2. Materials and Methods

### 2.1. Sample Preparation and Characterization

Commercial aluminum powder (purity: 99.8%, Riedel-de Haën, Seelze, Germany) with a particle size under 40 µm was used. The elemental composition was determined via X-ray Fluorescence (XRF S8-Tiger, Bruker, Bremen, Germany) and is summarized in Table 1. Mechanical alloying was performed in an air atmosphere using a high-energy planetary ball mill (Pulverisette P-7, Fritsch, Idar-Oberstein, Germany). Milling was conducted at 500 rpm with a ball-to-powder weight ratio of 2:1 using 12 mm diameter steel balls. To minimize overheating, cycles of 10 min milling followed by 5 min pauses were used, totaling 46 h of effective milling time.

The unmilled and milled powders were stocked for several days under an air atmosphere. The oxygen and nitrogen concentrations in the powders were measured using a LECO ONH836 elemental analyzer (LECO Corporation, St. Joseph, MI, USA). The results are presented in Table 2.

**Figure 1:**
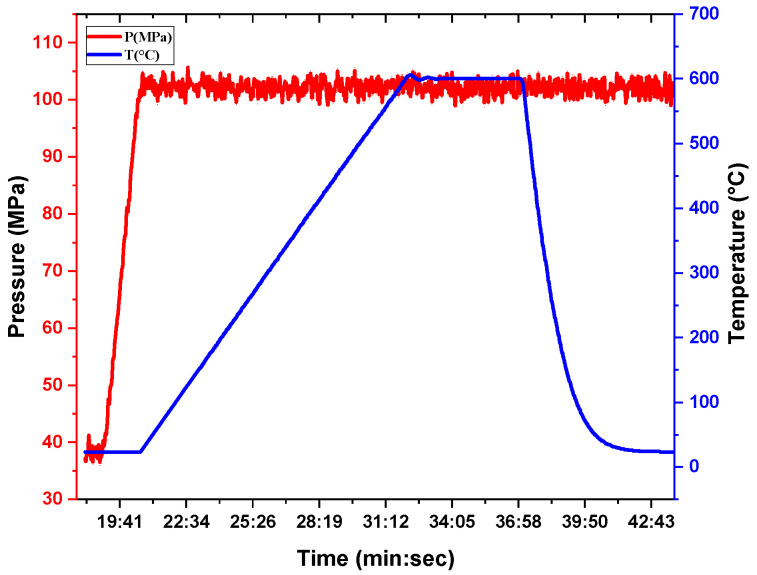
Example of SPS cycle parameters used for sintering at 600 °C and 100 MPa.

The milled powders were consolidated using spark plasma sintering (SPS) in a HPD 10 device (FCT Systeme GmbH, Frankenblick, Germany). Approximately 1 g of powder was introduced into a 10 mm diameter graphite die, with graphite foil placed between the die wall and powder to prevent sticking and reduce die wear. Sintering was carried out under uniaxial pressures of 50 MPa or 100 MPa. The temperature was monitored with a thermocouple placed 2 mm from the inner die wall. The heating ramp was 50 °C/min. The sintering temperature plateau was 550 °C, 600 °C, and 625 °C. The temperature was held for 5 min, followed by cooling at 50 °C/min. Pressure was maintained constant throughout the heating, dwell, and cooling phases. An example of the sintering cycle is presented in Figure 1. The resulting sintered samples were disks approximately 4 mm high after removal of the surface contaminated by the carbon foil.

The sintered samples were polished using SiC abrasive papers (up to P1200 grit), followed by super-finishing up to 30 nm colloidal silica. Residual polishing debris were removed via ultrasonic cleaning in ethanol for 5 min. Vickers microhardness (HV) testing was conducted using a 200 g load with a 10 s dwell time. A minimum of nine indentations were made to obtain a representative average hardness and a standard deviation value. Nanoindentation tests were performed using the high-precision, high-speed nanoindenter FT-I04 (Oxford Instruments-FemtoTools, Zurich, Switzerland) employing the Continuous Stiffness Method (CSM). The density of the SPS-consolidated samples was measured by Archimede’s method using the triple weighting method. The relative density was calculated as the ratio of experimental density to the theoretical density of aluminum 2.7 g/cm^3^.

XRD analysis of the powders and SPS-consolidated samples was carried out at room temperature using a Bruker D8 DISCOVER diffractometer (Bruker, Billerica, MA, USA) equipped with a LYNXEYE detector (Bruker, Billerica, MA, USA) and Cu-Kα radiation (λ = 1.5406 Å). Data were collected in θ–2θ mode from 20° to 90° (2θ) with a step size of 0.00204°. Phase identification was performed using EVA V7 software and the PDF database (ICDD, pdf 5+). Lattice parameters, crystallite size, and microstrain were extracted via Rietveld refinement using MAUD version 2.996 software [25,26,27]. Dislocation density (ρ) was evaluated using the following [28]:

(1) $$\rho =\frac{\left(2\sqrt{3}\times \varepsilon \right)}{\left(D\times b\right)}$$

where ε is microstrain, *D* is the crystallite size, and *b* is the Burgers vector, taken as $\frac{a}{\sqrt{2}}$ for CFC aluminum.

The morphology of powders and the microstructures of sintered samples were examined by scanning electron microscopy using JEOL JSM 7600F (JEOL, Tokyo, Japan). Particle size distribution was determined using a laser particle size analyzer (MALVERN-Panalitycal, Malvern, UK).

### 2.2. Tribological Tests

Dry sliding wear tests were performed using a custom-made ball-on-plate tribometer. Alumina balls (5 mm diameter) were used as counter faces under reciprocating linear movement with a displacement stroke of ±1 mm at 1 Hz. A constant normal load of 1 N was applied for 5000 cycles using dead weight. The relative ball/plate displacement during testing was monitored by a linear variable differential transformer (LVDT) sensor. For each friction cycle, an average friction coefficient (μ) is calculated from an energetic point of view via Equation (2):

(2) $$\mu =\frac{1}{2\times ∆h_{0}}\int \frac{\left|F_{T}\right|}{F_{N}}dh$$

where $∆h_{0}$, very close to 2 mm, refers to the length between the two positions of the ball corresponding to the tangential force $F_{T}=0$ and the normal force $F_{N}=1\;N$ in our case.

The topography of the surface of each friction track (plates and balls) was measured through a variable-focus optical microscope (Infinite Focus Alicona Imaging GmbH, Graz, Austria). The 3D surface topography data were analyzed thanks to Gwyddion free software version 2.68 [29] to measure roughness and calculate wear volumes. The wear volume is determined by adding the positive and the negative volumes with respect to the height of the original sample surface. The wear rate (µm^3^/mm) of the plate ($W_{P}$) and the ball ($W_{b}$) were then calculated as the wear volume normalized by units of sliding distance recovered by each sample, as defined by Equations (3) and (4), respectively, using the same method more precisely described in [30]:

(3) $$W_{P}=\frac{V_{P}}{2\times {∆h}_{0}\times N_{cycles}}$$

(4) $$W_{b}=\frac{V_{b}}{2\times {∆h}_{0}\times N_{cycles}}$$

where $V_{P}$ (µm^3^) and $V_{b}$ (µm^3^) are the wear volumes of the plate and the ball, respectively, and ${∆h}_{0}$ is the length of the friction track analyzed to obtain the wear volume of the plate.

## 3. Results and Discussions

### 3.1. Microstructure of Powders

**Figure 2:**
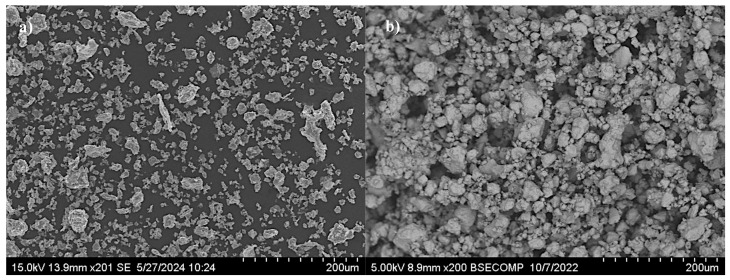
SEM images of aluminum powders: (**a**) unmilled Al, (**b**) Al milled for 46 h.

Secondary electron (SE) images of the unmilled aluminum powder and the powder milled for 46 h are presented in Figure 2. As shown in Figure 2a, the as-received powder particles exhibit relatively uniform morphology and size. In contrast, Figure 2b reveals significant morphological changes after 46 h of high-energy ball milling. The formation of larger particles is evident, attributed to cold welding and plastic deformation resulting from repeated ball–powder collisions.

**Figure 3:**
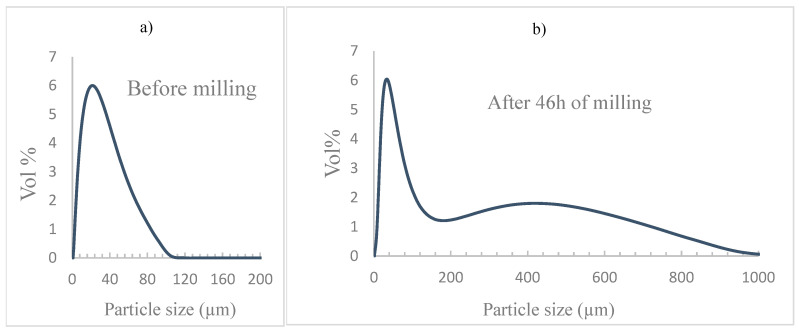
Grain size distribution: (**a**) unmilled Al, (**b**) Al milled for 46 h.

The corresponding particle size distributions are displayed in Figure 3. The volume-weighted mean particle size of the unmilled powder is approximately 21 µm; Figure 3a. After prolonged milling, seen in Figure 3b, the mean particle size increases markedly to 97 µm, consistent with SEM observations. The distribution profiles reveal that the highest volume percentage occurs at particle sizes of 22.9 µm and 34.7 µm for the unmilled and milled powders, respectively. Furthermore, particles below 10 µm constitute approximately 31.4% of the total volume in the unmilled sample, while this fraction decreases sharply to only 6.5% after 46 h of milling. These findings are consistent with those reported by Saboor Bagherzadeh et al. [31]. Despite some particle fracturing, cold welding and agglomeration are the dominant mechanisms during the high-energy milling of ductile metals.

### 3.2. XRD Analysis of Powders

**Figure 4:**
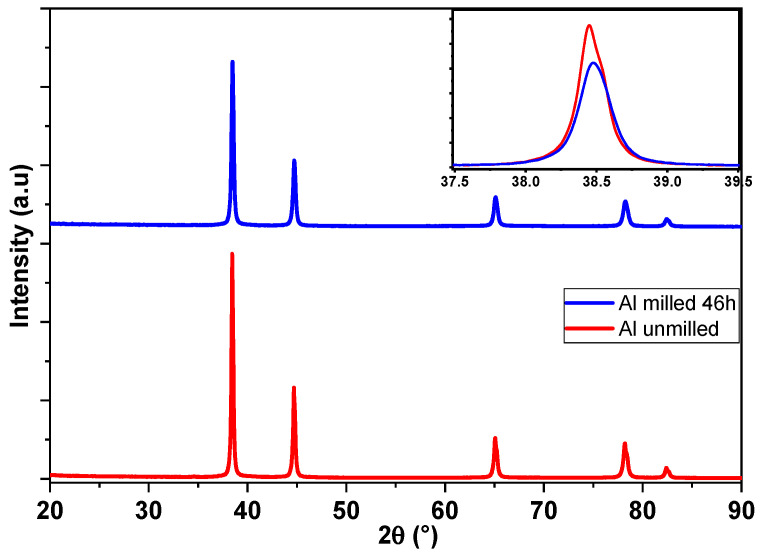
X-ray analyses of unmilled and milled Al powders with a full scan and a zoomed region in the inset showing the effect of milling on the maximum position and intensity for the Al phase.

The X-ray diffraction (XRD) patterns of the unmilled and 46 h milled aluminum powders are presented in Figure 4. Both diffraction patterns display peaks corresponding exclusively to the face-centered cubic (FCC) phase of aluminum, confirming the high purity of the powders. No additional peaks attributable to aluminum oxide (Al_2_O_3_) were observed, even though the high-energy ball milling process was led under an air atmosphere.

A comparison of the diffraction profiles reveals that the peak intensities of the milled powders are significantly reduced relative to the unmilled powders, leading to an increase in peak width, which indicates microstructural changes such as grain refinement and increased lattice defects. Moreover, a noticeable dissymmetry and slight shift in the principal (111) Al peak towards higher 2θ angles is observed after 46 h of milling. The shift in the main peak, highlighted in the inset of Figure 4, suggests a decrease in the lattice parameter, probably due to the introduction of residual stress during the high-energy ball milling process [32].

These results are consistent with microstructural evolution mechanisms commonly associated with mechanical alloying, including grain size reduction, accumulation of lattice strain, and generation of crystalline defects.

**Figure 5:**
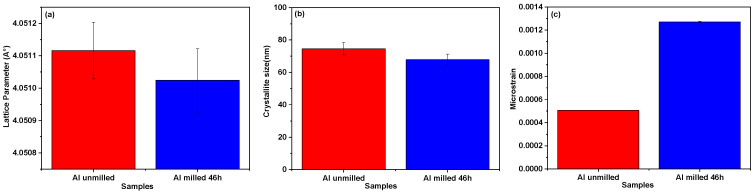
Variations in lattice parameter (**a**), crystallite size (**b**), and lattice strain (**c**) of unmilled and milled Al powders.

Figure 5 presents the evolution of the lattice parameter, crystallite size, and lattice microstrain of aluminum powders as a function of milling time, extracted via Rietveld refinement using MAUD software. As shown in Figure 5a, a slight decrease in the lattice parameter is observed after 46 h of milling. However, the variation remains within the experimental error margins, indicating that the change is not statistically significant. The crystallite size, depicted in Figure 5b, exhibits a modest reduction from 74 nm unmilled powders to 68 nm after 46 h of milling. This limited decrease suggests minimal crystallite fragmentation during the milling process. Similar findings were reported by Salur et al. [33,34], who observed a reduction in crystallite size of unmilled pure aluminum AA7075 and AA7075-0.5 wt% Y_2_O_3_ milled 10 h from 35 nm to 25 nm.

Figure 5c shows that the lattice microstrain increases by approximately 2.5 times as a milling effect. This behavior is consistent with the generation of internal stress and structural defects induced by repetitive impacts during high-energy ball milling. Table 3 lists the corresponding dislocation densities, estimated using Equation (1). A notable increase in dislocation density is observed in the sample milled for 46 h, confirming the effect of prolonged mechanical processing on the defect structure of the aluminum powders. Tousi et al. [35] have also reported that the decrease in crystallite size indicates a high dislocation density due to excessive and repetitive collisions between balls, powder, and jar. Both lattice deformation and dislocation density have increased due to lattice distortions, leading to local plastic deformation during milling.

### 3.3. DSC of the Unmilled and Milled Powders

**Figure 6:**
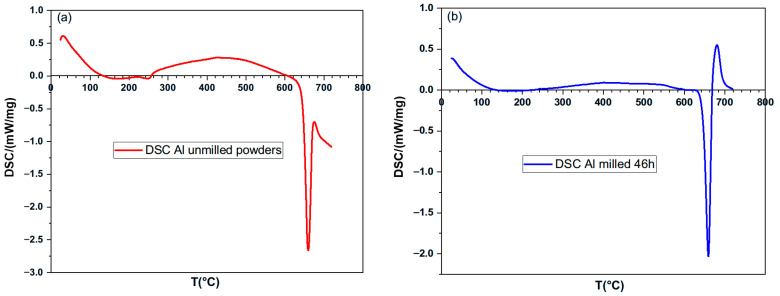
Scanning calorimetric differential rising to the melting point: (**a**) Al unmilled powders and (**b**) Al milled for 46 h.

To explore the possibility of partial melting below the standard melting point of pure aluminum (660.4 °C), Differential Scanning Calorimetry (DSC) was conducted. Figure 6 presents the results of DSC for both unmilled and 46 h milled powders. A melting peak is observed at 646 °C, below the theoretical melting point. Additionally, the reaction enthalpy is significantly reduced compared to that of pure aluminum (394 J/g). Specifically, the unmilled powder shows enthalpy of (194.4 J/g), while the milled powders show an even lower value of about (110.6 J/g). With the powders being partially oxidized (7 at.% and 10.8 at.% of O in the unmilled and milled powders, respectively), the measured latent enthalpy of fusion may be lower than the theoretical value. However, in the present case, the difference is significant and cannot only be attributed to the presence of alumina in the Al particles. This difference can probably be mainly attributed to the accumulation of deformation energy in the distorted lattice caused by intense mechanical stress. These interpretations are consistent with the XRD results in Figure 5, which reveal small crystallite sizes and high microstrain values for both unmilled and milled powders.

These findings are crucial for an enhanced comprehension of the behavior of the powders during the high-temperature SPS process.

### 3.4. DRX of SPS-Consolidated Samples

**Figure 7:**
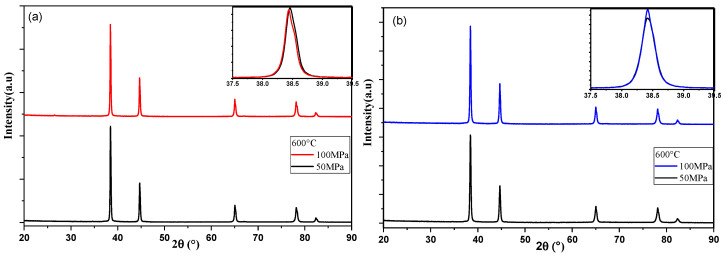

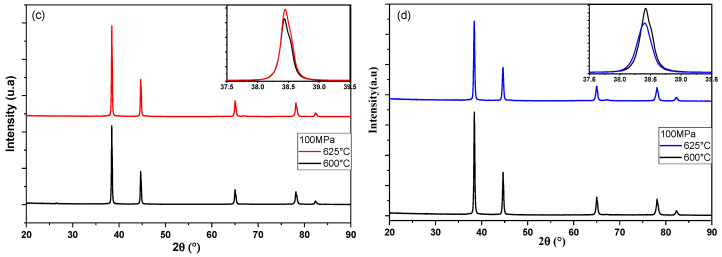
(**a**–**d**): influence of the SPS pressure and temperature, respectively, on the XRD patterns of consolidated samples with peak zoom (111); (**a**,**c**) unmilled Al powders; (**b**,**d**) milled Al powders for 46 h.

The XRD patterns of SPS-consolidated samples from Al powders both unmilled and milled are shown in Figure 7 as a function of the sintering pressure and temperature. The XRD patterns show that the only phase detected is pure aluminum. Zooming in on the main peak (111) under various consolidation conditions reveals the influence of SPS parameters on peak position and broadening.

**Figure 8:**
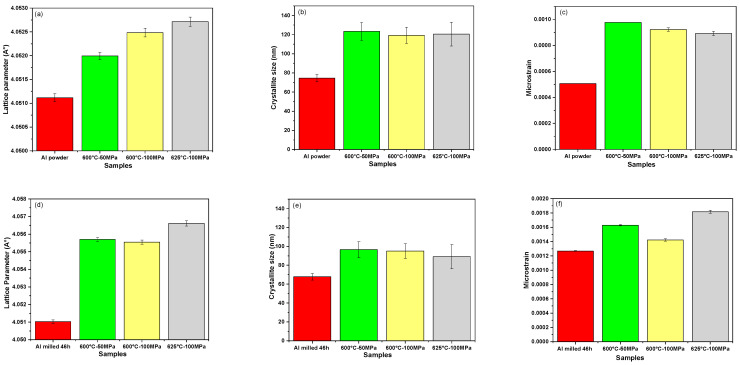
Crystallographic parameters for SPS-consolidated powders. (**a**–**c**) Al raw unmilled powder and (**d**–**f**) Al raw milled powder for 46 h: (**a**,**d**) lattice parameter; (**b**,**e**) crystallite size; and (**c**,**f**) microstrain.

For the consolidated unmilled powder (see Figure 7a), increasing the pressure from 50 MPa to 100 MPa results in a slight peak shift to a smaller 2θ angle. This shift indicates a change in lattice parameter, which leads to a slight increase from 4.0519 Å ($\pm 1\times 10^{-4})$ to 4.0524 Å ($\pm 1\times 10^{-4}$) as obtained by the Rietveld analysis; Figure 8a. The changes are also accompanied by a modest reduction in crystallite size and microstrain.

For the milled powders (see Figure 7b), the consolidation at 600 °C under increasing pressure from 50 MPa to 100 MPa involves a minor increase in peak intensity without any noticeable peak intensity displacement. Interestingly, this is accompanied by a reduction in microstrain as shown in Figure 8f, which can be assigned to the milling effect.

We can conclude that the increase in the SPS pressure has no significant influence in the Rietveld parameters of both samples made of unmilled and milled powders. The influence of sintering temperature is revealed by Figure 7c, which shows that increasing the temperature from 600 °C to 625 °C for the unmilled Al results in a decrease in peak width with a slight peak shift to a smaller 2θ angle. This suggests a slight reduction in the lattice strain, as reflected in Figure 8c.

In contrast, Figure 7d shows that for the milled powders, raising the SPS temperature from 600 °C to 625 °C results in a subtle shift towards lower 2θ values and an increase in peak width. This reflects an increase in microstrain (see Figure 8f).

The evolution of the crystallographic parameters obtained by Rietveld refinement of SPS-consolidated unmilled and milled Al powders XRD patterns reveals that sintering slightly increases microstrain, the crystallite size and the lattice parameter. These findings are consistent with the dislocation density (ρ) values reported in Table 3, which were calculated using Equation (1). It can be concluded that increasing the sintering temperature increased the dislocation density in the milled samples, likely due to the presence of oxides blocking the mobility of dislocations and preventing defect recovery, whereas it leads to a decrease in dislocation density in unmilled samples. Similar findings have been reported by Soares et al. [36], who reported that the dislocation density of the (AA7075) aluminum alloy milled for 1 h and consolidated by SPS at 600 °C–100 MPa for 15 min is four times higher than the unmilled SPS-consolidated sample. Additionally for all parameters of sintering used, it can be noted that the dislocation density of the SPS milled samples is always higher than the SPS unmilled samples, which is supposed to produce enhanced hardness and mechanical strength (addressed below).

### 3.5. Microstructure of SPS-Consolidated Pellets

Prior to analyzing the microstructure, we quantified the oxygen and nitrogen elemental composition of the samples consolidated by SPS at 600 °C and 100 MPa using unmilled and milled powders. The measurements were performed with a LECO ONH836 elemental analyzer, and the results are presented in Table 4. By comparing these values with those in Table 2, it can be observed that the SPS process does not lead to an increase in the overall oxygen content.

**Figure 9:**
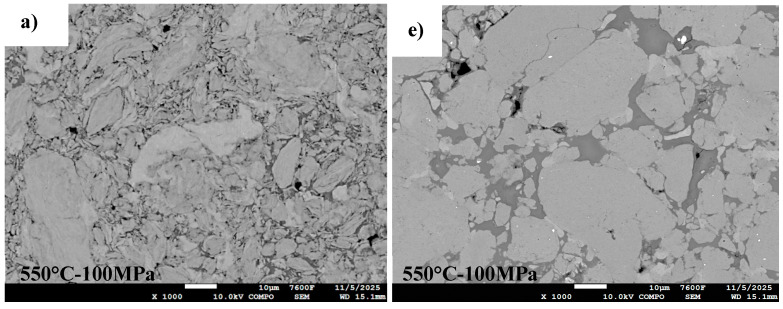

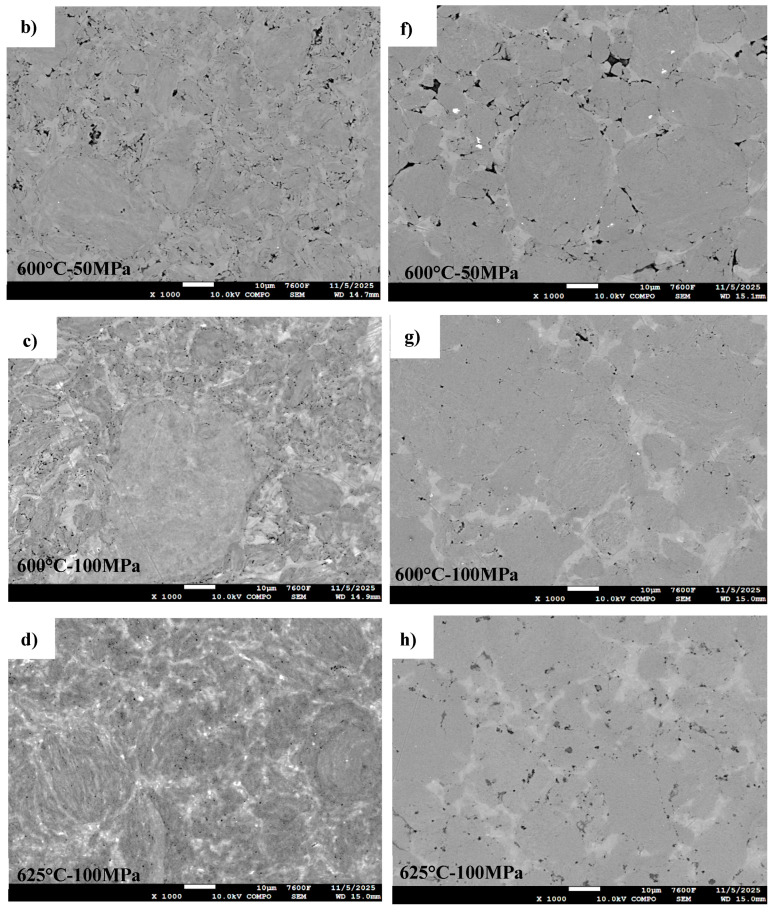
SEM images of SPS-consolidated samples using various powders: (**a**–**d**) Al unmilled powders, (**e**–**h**) high-energy ball milled for 46 h and SPS-consolidated at 550 °C–50 MPa, 600 °C–50 MPa, 600 °C–100 MPa, and 625 °C–100 MPa, respectively.

Figure 9 illustrates the SEM images of unmilled and milled Al powders consolidated by SPS at varying temperatures (550, 600, and 625 °C) under two pressure conditions (50 MPa and 100 MPa). Visible porosities are observed within the microstructure of the consolidated compact at 550 °C–50 MPa (Figure 9a,e). However, increasing the sintering temperature to 600 °C–50 MPa at the same pressure significantly reduces the porosity, and a further improvement in densification is achieved at 600 °C and 100 MPa, where pores are effectively absent. These observations are consistent with previous studies [37], which highlight the positive influence of elevated sintering temperatures on densification behavior.

The densification trend is quantitatively presented by the data in Table 5, which includes processing parameters and the corresponding relative densities of the sintered pellets. An increase in both sintering temperature and applied pressure leads to higher relative densities, approaching the theoretical density of the aluminum, indicating that the samples have been fully consolidated by SPS, which are similar behaviors observed by other researchers [38].

The relative density values indicate that full densification (100%) was achieved for the milled aluminum sample consolidated at 600 °C–100 MPa. The unmilled SPS sample sintered under the same conditions showed a relative density of 98%. Full densification was achieved at 625 °C for the unmilled sample. We must mention that in this case, the SPS consolidation curve recorded during the process suggests localized melting, which can have a detrimental impact on the mechanical properties of the sintered pellet. This is consistent with the work of Liu et al. [21], who reported a sharp decrease in microhardness when pure aluminum was consolidated by SPS at 600 °C with a holding pressure of 300 MPa for 6 min, attributing it to grain coarsening and localized melting. These phenomena compromise the mechanical strengthening effects associated with nanostructured grains.

In comparison, the SPS-milled aluminum sample consolidated at these higher parameters of 625 °C–100 MPa exhibited a decrease in relative density (98.7%), associated with an occurrence of black dots (Figure 9h), which are probably Al_2_O_3_ oxides, as further suggested by EDS mappings and nanoindentation tests on these zones.

**Figure 10:**
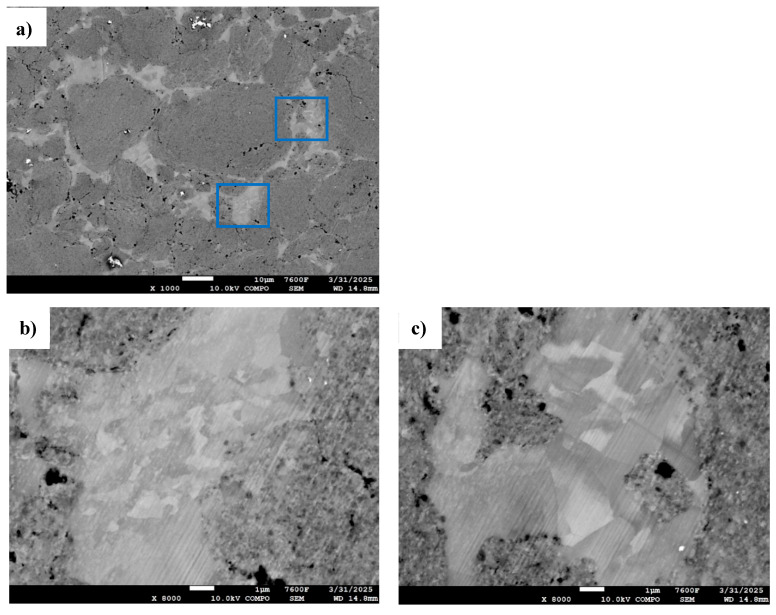
SEM images of (**a**) Al powders milled for 46 h and consolidated at 600 °C–100 MPa and magnification on the two blue-squared regions (**b**,**c**).

All micrographs of consolidated samples reveal two distinct regions: dark-gray, round-shaped particles and light-gray, angular zones filling the inter-particle voids. These features are clearly visible in Figure 10, captured at the sample core. Figure 10a shows a SEM image of the consolidated Al powders milled for 46 h at 600 °C and 100 MPa. The magnified views in Figure 10a,c emphasize the two different microstructures: the dark-gray regions consisting of ultra-fine grains (below 100 nm) and the light-gray zones containing coarser grains (>1 µm). These larger grains are indicative of recrystallization, as confirmed by their irregular shapes, a known feature of such processes. Other researchers noticed heterogeneity between grains; Guyon et al. [39] observed finer grains in the interparticle zones of TiAl, while Saviot et al. [40] found larger grains in interparticle zones on HEA compounds consolidated from mixtures of co-milled elemental powders.

Two hypotheses are proposed for the formation of the light-gray, coarse-grained regions. The first is a localized melting, followed by solidification and recrystallization during the SPS process as proposed by Zuniga et al. [41], who suggest that this occurs due to high current density at particle contact points early in the process, prior to full densification. The second hypothesis [40] postulates that these zones result purely from recrystallization and grain growth, facilitated by the internal energy stored in deformed milled grains, without involving localized melting. In both cases, these light-gray zones should exhibit low oxygen contents and low hardness values.

**Figure 11:**
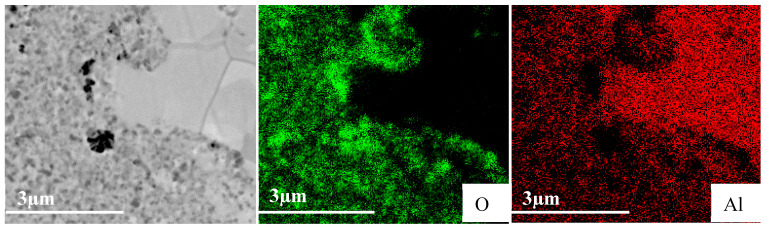
SEM elemental mapping of Al sintered at 625 °C–100 °C made from milled powders.

To clarify the composition of these regions, EDS measurements were performed on the SPS 625 °C–100 MPa sample made from milled powders (Figure 11). Three distinct zones can be observed: a light-gray zone with large grains, a dark-gray zone with small nano-grains, and small black spots. The light-gray zone, which contains large grains, exhibits low oxygen content (2 at.%) and high aluminum content (98 at.%), supporting the conclusion that it corresponds to recrystallized aluminum. The dark-gray zones are composed of fine grains and show an average oxygen content of about (26 at.%), suggesting that this region is a mixture of aluminum and aluminum oxides. The black spots display high oxygen content (44 at.%) and low aluminum content (56 at.%), indicating that these spots are highly enriched in oxides. Moreover, one must mention that the dimension of these spots (<0.6 µm) is small compared with the volume investigated by EDS (~1 µm^3^), meaning that the measurements are highly influenced by the surrounding dark-gray zone. Thus, the real oxygen content of the black spots is probably much higher than the measured content. These black dots are then probably formed mainly of oxides.

### 3.6. Nanoindentation

**Figure 12:**
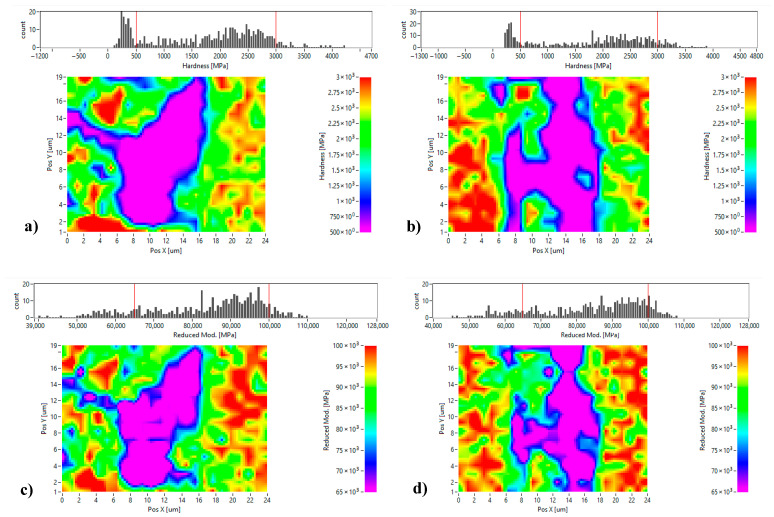
Nanoindentation results: (**a**,**c**) nanohardness and reduced elastic modulus mappings of the region presented in Figure 10b; (**b**,**d**) nanohardness and reduced elastic modulus mappings of the region presented in Figure 10c.

To further assess the mechanical characteristics of each phase as previously discussed, nanoindentation tests were carried out (Figure 12). We proceeded to investigate by nanoindentation the values of nanohardness and reduced elastic modulus of the exact same zones presented in the SEM images of Figure 10b,c. The light-gray zones exhibited low nanohardness values of ~250 MPa. In contrast, the dark-gray zones displayed significantly higher hardness values of ~3055 MPa, which can be attributed to their fine microstructure and higher oxygen content detected by EDS.

Nanohardness measurements were made on all the SPS-consolidated samples. The quantitative results are summarized in Table 6 and Table 7 obtained by randomly selecting 40 values by zone (light-gray and dark-gray zones). We used the analysis of variance test (ANOVA) for statistical validation of the nanoindentation observations. The following information can be drawn:

- Both zones (dark-gray and light-gray) exhibit significantly higher values of hardness and elastic modulus on samples obtained by consolidation of milled powders compared to those consolidated from unmilled powders for all same SPS parameters.
- For the SPS-consolidated unmilled powders:
  ○ In the dark-gray zones (nano-grains), the nanohardness average values are not statistically different.
  ○ In the light-gray zones (recrystallized grains), the nanohardness average values are statistically different with a confidence index of >99%
- For SPS-consolidated milled powders:
  ○ In the dark-gray zones (nano-grains), the nanohardness average values are statistically different with a confidence index >99%.
  ○ In the light-gray zones (recrystallized grains), the nanohardness average value of the SPS 600 °C–50 MPa sample is statistically different with a confidence index >99% in comparison with the other two samples, while the values measured on samples SPS 600 °C–100 MPa and SPS 625 °C–100 MPa are not statistically different.
- The average reduced elastic modulus of both zones (nano-grains or recrystallized grains) is bigger for samples made of milled powders than those made of unmilled powders.

**Figure 13:**
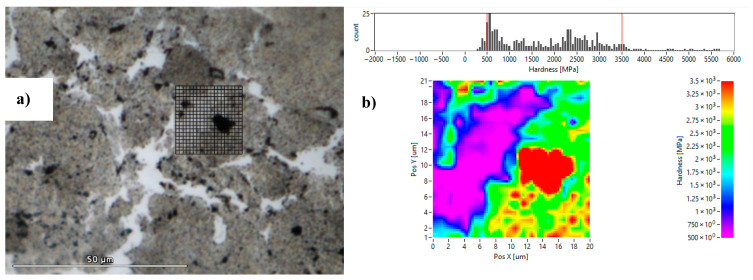
Nanoindentation results of Al milled 46 h consolidated sample at 625 °C–100 MPa: (**a**) optical microscopy and fine-scale nanoindentations grid on a region presenting black zones and (**b**) corresponding nanohardness cartography.

On the SPS-consolidated samples produced from milled powders, a minor phase consisting of dark dots can be observed in Figure 13a, which are likely aluminum oxides. This is confirmed by finer nanoindentation measurements made on these black spots observed in Figure 13a, revealing an exceptionally high nanohardness value of 4027 MPa. This high value can be explained to the combined effects of the microstructural refinements and the formation of oxide-rich domains (dark dots). These results prove that the milling process of the powders enhances considerably the material’s hardness obtained after SPS consolidation.

Under all processing conditions, milled samples consistently exhibit higher nanohardness and elastic modulus values than their unmilled samples, with the most marked variations observed in the dark-gray phases. This trend is in line with observations made in similar studies, confirming the effect of milling in enhancing the indentation modulus [32].

These nanoindentation results highlight the mechanical contrast between the different phases. However, to fully assess the mechanical performance of the consolidated samples, additional microhardness measurements are necessary.

### 3.7. Microhardness

**Figure 14:**
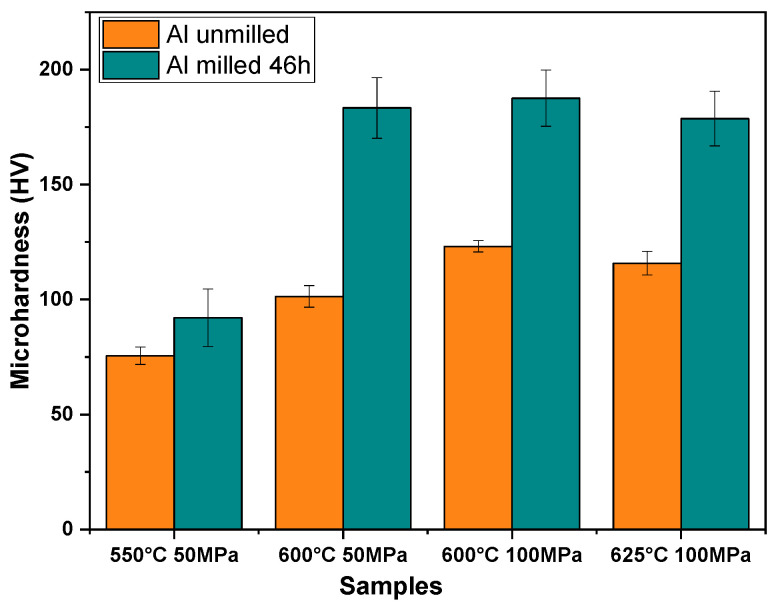
Effect of sintering temperature and pressure on microhardness on the aluminum unmilled and milled for 46 h.

Figure 14 presents the influence of sintering temperature and pressure on the microhardness of aluminum samples consolidated via the SPS process. The most obvious observation is that the samples made from milled powders exhibit significantly higher values of microhardness compared to the unmilled powders processed using the same SPS parameters.

Figure 14 reveals that at a low consolidation pressure of 50 MPa, microhardness increases with increasing sintering temperature from 550 °C to 600 °C, which can be easily explained by the poor level of densification at a low temperature.

For temperatures higher or equal to 600 °C, the ANOVA reveals a statistically representative influence of the pressure as well as of the temperature on the microhardness of the consolidated samples made from unmilled powders. On the contrary, the ANOVA shows that there is no statistically representative influence of pressure or temperature on the microhardness values of the SPS samples made from milled powders.

The difference between the samples obtained from unmilled and milled powders reflect the differences in crystallite size, microstrain, and the density of defects introduced during milling. The plastic deformation can be achieved by dislocation displacements, sliding or twinning [17,42]. In this case, the hardening can be clearly attributed to grain refinement (Hall–Petch effect) [43] and to the high density of defects (Williamson–Hall effect) [6]. Both grain refinement and the high density of defects shorten the displacement of dislocations and slidings. The porosity observed after low-temperature SPS processing lead to easier collapse under external force, resulting in reduced microhardness [44,45].

### 3.8. Tribological Characterizations

The consolidated samples were submitted to tribological tests to characterize the influence of the SPS parameters on the coefficient of friction and on the wear rate. Aluminum generally presents mainly adhesive wear mechanisms. The local heating and the presence of oxygen favor the production of an alumina thin layer on the friction track. This alumina is brittle and can generate abrasive wear when oxide particles detach from the wear track. This can produce an increase in the coefficient of friction during tribological tests.

#### 3.8.1. Coefficient of Friction

**Figure 15:**
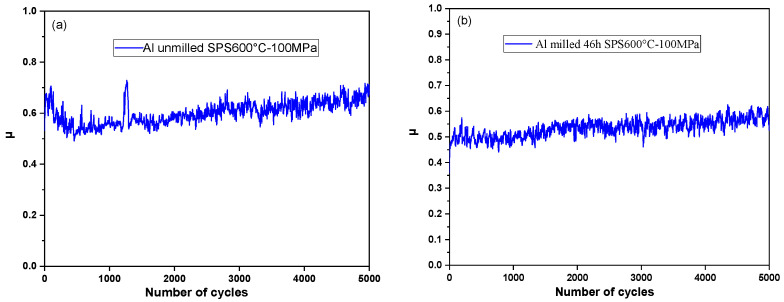
Coefficient of friction of the SPS samples consolidated at 600 °C–100 MPa; (**a**) Al unmilled powders; (**b**) Al 46 h milled powders.

Figure 15 depicts the evolution of the friction coefficient throughout the tribological test for SPS-consolidated samples obtained with unmilled and milled powders. Three tests were made for each sample, and high reproducibility was observed. For all the samples, the coefficient of friction increases slightly with the number of cycles, as illustrated in Figure 15. For comparison purposes between samples, and to avoid considering the running-in effect, the average value of the coefficient of friction ($\mu_{i}$) between 3000 and 5000 cycles has been considered. Table 8 shows the values of $\mu_{i}$ for all the samples. The results indicate that the mechanical milling significantly reduces the $\mu_{i}$ values for all sintered samples made of milled powders. This feature is attributed to the increase in hardness (see Figure 14) produced by the grain refinement and the high density of defects introduced by the milling. The density of the sintered samples (see Table 5) can also have an influence on this decrease. The sample milled for 46 h and consolidated at SPS 600 °C–100 MPa exhibits the lowest value of $\mu_{i}\;$= 0.56, and we showed previously that the same sample presented the highest microhardness value and reached full densification (100%).

It is also worth noting that the SPS consolidation parameters have no significant effect on the coefficient of friction except for the unmilled powders consolidated at 625 °C–100 MPa (which achieves the highest value of the ($\mu_{i}=0.88$). This is the same sample that showed partial fusion during the SPS process and also exhibited the lowest hardness of the unmilled powders consolidated samples.

Rengifo et al. [23] found a friction coefficient of 0.87 for unmilled aluminum powders consolidated by SPS at 500 °C–50 MPa for 10 min in tribological test conditions very close to ours. This value is very close to that obtained in our work ($\mu_{i}=0.88$) but under higher SPS conditions of 625 °C and 100 MPa using unmilled powders, too. We point out that our powders were exposed to air, which provoked partial oxidation. Also, the samples SPS-consolidated at 625 °C and 100 MPa suffered partial fusion during the SPS process, as mentioned earlier. This could explain why the value of the coefficient of friction of this sample is close to Rengifo’s result. The value of the coefficient of friction of the sample consolidated by SPS at 600 °C and 50 MPa or 100 MPa are substantially lower, reaching 0.65 and 0.67, respectively.

Furthermore, Mohammed et al. [22] reported a friction coefficient of 0.85 for aluminum powders milled for 24 h and consolidated by SPS at 550 °C–50 MPa for 10 min, which is greater than all the coefficients obtained in our study, regardless of milling duration or SPS parameters. We must mention that our test conditions are different (alumina balls in our case and stainless steel balls in their case).

#### 3.8.2. Wear Rate

**Figure 16:**
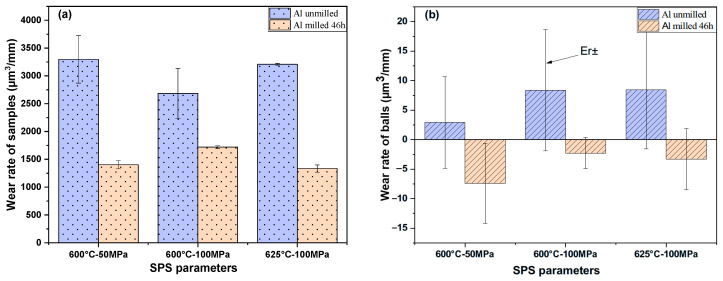
Average wear rate (**a**) of plate samples and (**b**) of balls.

Figure 16 shows the average wear rate for all the samples. The wear rate of plates $W_{P}$ was determined using Equation (3). According to Figure 16a, the $W_{P}$ of the samples after milling decreased significantly, almost to half the value of unmilled samples. The lower $(W_{P}$) is observed for the milled sample consolidated at 625 °C–100 MPa, which is 58% lower than the value of the sample obtained out of unmilled powders with the same SPS parameters.

In addition, as demonstrated in Figure 16b, the wear rate values of the balls $W_{b}$, calculated using Equation (4), are close to zero. The $W_{b}$ values of the samples obtained with unmilled powders are positive, which denotes a mass loss of the balls. Interestingly, the average values of $W_{b}$ are negative for all the samples obtained from milled powders, which denotes that the transfer of mass from the samples to the counter face balls is superior to the mass loss of the balls.

It can be concluded that milling has a major impact on improving the wear behavior of our samples, which is associated with higher hardness values. This result is consistent with similar studies: Abdollahi et al. [46] showed that the wear rate of nanostructured Al2024 alloy milled for 50 h and consolidated by hot extrusion, under a load of 20 N and a sliding distance of 3000 m, is 30% lower than that of the unmilled sample, since the increased of the aluminum alloy hardness reduces the wear rate due to grain refinement. Similarly, Jafari et al. [47] studied the wear rate of unmilled aluminum consolidated by SPS at 560 °C and 50 MPa for 5 min. They reported a wear rate of $390\frac{\mu m^{3}}{mm}$ using 5 mm diameter pin-on-disk tests under a normal load of 1 N and 500 m of sliding distance with 0.07 m/s linear speed. It is worth mentioning that the values obtained in the present paper are almost ten times bigger than those obtained by Jafari et al. The difference can easily be explained by the differences in counter face shape and nature (4 mm diameter alumina ball for our tests and 5 mm diameter steel cylinders for Jafari et al.), which, in our case, about hundred-times-larger contact pressures.

#### 3.8.3. Morphologies of the Worn Surface

In pure aluminum, the most common mechanism wear is adhesive wear [48], which occurs when two surfaces, alumina ball and sample, come into contact and slide against each other.

**Figure 17:**
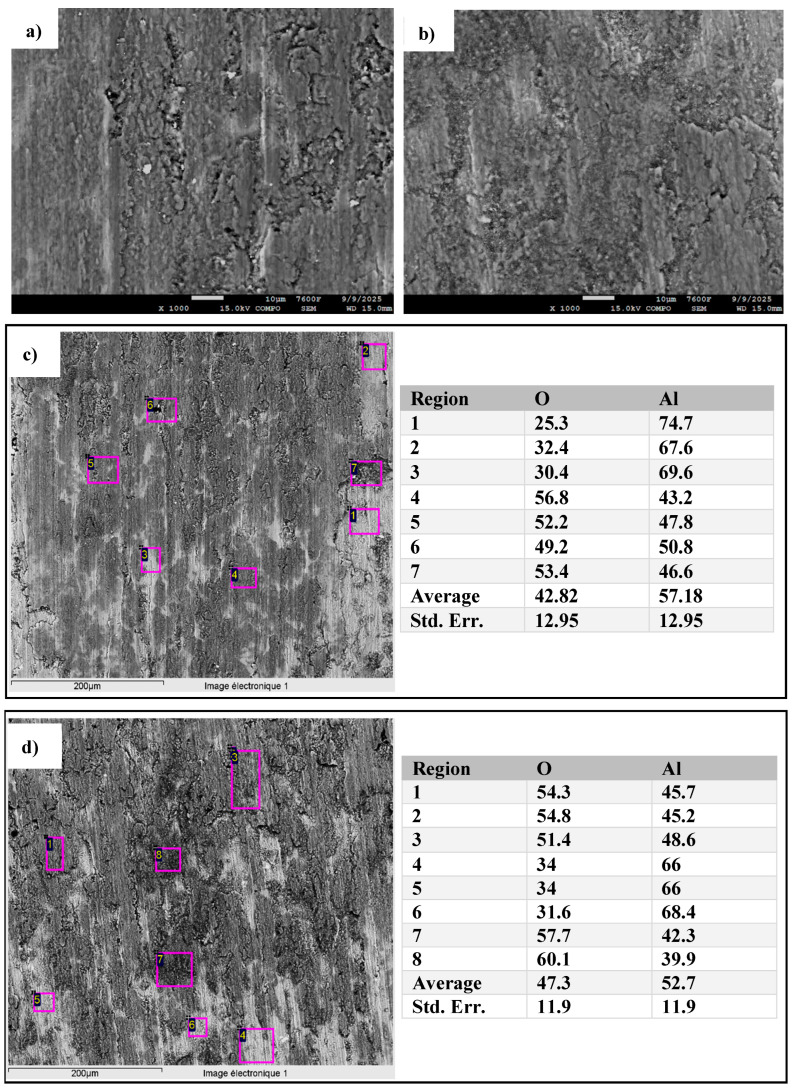
SEM images of the worn surface of the SPS-consolidated sample at 600 °C–100 MPa; (**a**): unmilled Al and (**b**): milled Al 46 h, (**c**,**d**) EDS analyses of the selected areas for the unmilled and milled sample, respectively.

Figure 17 illustrates SEM observations and EDS analyses of the friction track of the SPS-consolidated aluminum both unmilled (Figure 17a) and milled for 46 h (Figure 17b) and sintered at 600 °C–100 MPa after 5000 wear cycles. Figure 17a also shows regions subject to abrasive wear on the samples consolidated from unmilled powders, as regions where superficial grains of material were torn out from the surface. Localized heating and oxidation of the surface promote the formation of an Al_2_O_3_ layer. This oxide layer is intrinsically brittle and undergoes three-bodies abrasive wear. The hard, brittle Al_2_O_3_ phase tends to generate relatively large wear debris particles, which, in turn, increases both the wear rate and coefficient of friction. This assumption is consistent with the SEM micrographs shown in Figure 17. Thus, the worn surface of the sample made of unmilled powders (Figure 17a) shows numerous wide grooves, large wear debris accumulated on the friction track, material delamination, and localized plastic deformation, characteristic of a combined abrasive and adhesive wear mechanism.

In contrast, the milled powders consolidated sample (Figure 17b) shows a notably reduced amount of wear, characterized by finer scratches, limited flaking, and less pronounced plastic deformation.

EDS analysis of the worn surface (Figure 17c,d) indicates high oxygen content in both the unmilled and milled samples, with average values of 42.82% and 47.3%, respectively, but a large dispersion of results, with low oxygen content values as well as high values that suggest Al_2_O_3_.

In conclusion, milling improves wear resistance and reduces surface damage by minimizing the occurrence of abrasive wear.

## 4. Conclusions

This work investigated the effect of SPS consolidation parameters on high-energy ball milled aluminum powders. The powders were milled under an air atmosphere, with the intention of studying the implications of this milling process simplification. The impact of SPS parameters (temperature and pressure) on the structural, morphological, mechanical, and tribological properties are studied. Comparisons are made with SPS-consolidated unmilled powders.

Several conclusions can be drawn:

- In contrast to most studies in the literature where milling is achieved under an inert atmosphere (argon), this work was conducted in air. This particularity promotes the formation of alumina dispersion, which may contribute to the mechanical reinforcement of the consolidated materials.
- High-energy ball milling promoted powder agglomeration and increased grain size distribution while significantly refining crystallite size.
- The milled-aluminum sintered SPS sample achieved full densification (100%) at 600 °C and 100 MPa, which is not the case for the unmilled sample consolidated at these SPS parameters.
- All SPS-consolidated samples made of milled powders exhibited a higher dislocation density compared to the samples made of unmilled powders. This leads to greater hardness, as dislocation interactions restrict plastic deformation.
- Two principal morphologies were identified: small, agglomerated grains forming a hard phase, and recrystallized grains forming a soft phase.
- For the SPS temperature set to 625 °C, the unmilled and milled consolidated samples showed localized melting, whereas the sample consolidated from milled powders developed clusters of aluminum oxides.
- Milling has a measurable effect on improving the wear resistance of the samples, where the coefficient of friction varies from 0.68 for unmilled powders to 0.56 for milled powders after SPS consolidation at 600 °C – 100 MPa. The abrasive friction is minimized in samples made of milled powders.

The high-energy ball milling and SPS consolidation technique offers a cost-effective and rapid option for the manufacture of aluminum products with superior mechanical properties. This work provides insights into the processing of aluminum powders under non-inert conditions and can serve as a reference for future studies.

Works in progress explore the addition of hard reinforcement particles to further enhance the mechanical and tribological performance of the material and will use the results presented in this paper as references for comparison.

## Figures and Tables

**Table 1:** Elemental composition of the commercially aluminum powder.

Element	Al	Fe	Si	Ti	Zn	V	Ga	Ni	Cu	Mo	Zr
Concen-tration	99.8 at.%	0.11 at.%	363 ppm	120 ppm	106 ppm	101 ppm	64 ppm	59 ppm	42 ppm	18 ppm	15 ppm

**Table 2:** Oxygen and nitrogen elemental analysis of the air-exposed unmilled and milled powders.

Element	Al Unmilled Powders, at. %	Al Powders Milled for 46 h, at. %
O	7.0 (±0.6)	10.8 (±0.9)
N	0.017 (±0.006)	0.028 (±0.010)

**Table 3:** Dislocation density ρ (m^−2^) of powders and SPS samples as deduced from the XRD pattern.

Sample	ρ Unmilled (m^−2^)	ρ Milled for 46 h (m^−2^)
Powders	$8.23\times 10^{13}$	$2.27\times 10^{14}$
SPS600 °C–50 MPa	$9.58\times 10^{13}$	$2.05\times 10^{14}$
SPS600 °C–100 MPa	$9.37\times 10^{13}$	$1.81\times 10^{14}$
SPS625 °C–100 MPa	$8.96\times 10^{13}$	$2.48\times 10^{14}$

**Table 4:** Oxygen and nitrogen content of the SPS-consolidated unmilled and milled powders.

Element.	Al Unmilled Powders SPS600 °C–100 MPa, at. %	Al Milled for 46 h SPS600 °C–100 MPa, at. %
O	7.4 (±0.6)	9.9 (±0.8)
N	0.007 (±0.003)	0.033 (±0.004)

**Table 5:** Density of the sintered SPS samples.

Sample	Relative Density (%)
Al unmilled SPS 550 °C–50 MPa	82.1 (±0.9)
Al unmilled SPS 600 °C–50 MPa	95.4 (±0.9)
Al unmilled SPS 600 °C–100 MPa	98.0 (±0.6)
Al unmilled SPS 625 °C–100 MPa	100 (±0.5)
Al milled 46 h SPS 550 °C–50 MPa	81.6 (±0.5)
Al milled 46 h SPS 600 °C–50 MPa	95.5 (±0.4)
Al milled 46 h SPS 600 °C–100 MPa	100 (±0.9)
Al milled 46 h SPS 625 °C–100 MPa	98.7 (±0.6)

**Table 6:** Nanohardness results of SPS-consolidated samples.

Samples	Dark Gray Phase (MPa)	Light Gray Phase (MPa)
Al unmilled SPS 600 °C–50 MPa	2634 (±109)	150 (±29)
Al unmilled SPS 600 °C–100 MPa	2635 (±130)	380 (±60)
Al unmilled SPS 625 °C–100 MPa	2637 (±97)	480 (±28)
Al milled 46 h SPS 600 °C–50 MPa	2740 (±85)	400 (±65)
Al milled 46 h SPS 600 °C–100 MPa	3415 (±175)	662 (±78)
Al milled 46 h SPS 625 °C–100 MPa	4027 (±180)	630 (±73)

**Table 7:** Reduced elastic modulus results of SPS-consolidated samples.

Samples	Dark Gray Phase (MPa)	Light Gray Phase (MPa)
Al unmilled SPS 600 °C–50 MPa	93.5 (±5)	28.5 (±9)
Al unmilled SPS 600 °C–100 MPa	85 (±5)	61 (±8)
Al unmilled SPS 625 °C–100 MPa	95 (±5)	71 (±3)
Al milled 46 h SPS 600 °C–50 MPa	90 (±2)	65 (±5)
Al milled 46 h SPS 600 °C–100 MPa	101 (±4)	77 (±3)
Al milled 46 h SPS 625 °C–100 MPa	115 (±7)	76 (±4)

**Table 8:** The average coefficient of friction $\mu_{i}$ of all sintered samples.

Samples	$\mu_{i}$
Al unmilled SPS 600 °C–50 MPa	0.65 (±0.04)
Al unmilled SPS 600 °C–100 MPa	0.67 (±0.14)
Al unmilled SPS 625 °C–100 MPa	0.88 (±0.061)
Al milled 46 h SPS 600 °C–50 MPa	0.58 (±0.16)
Al milled 46 h SPS 600 °C–100 MPa	0.56 (±0.03)
Al milled 46 h SPS 625 °C–50 MPa	0.63 (±0.18)

## Data Availability

The raw data supporting the conclusions of this article will be made available by the authors on request.
